# Overexpression of miR-320-3p, miR-381-3p, and miR-27a-3p Suppresses Genes Related to Midline Facial Cleft in Mouse Cranial Neural Crest Cells

**DOI:** 10.3390/ijms262110730

**Published:** 2025-11-04

**Authors:** Chihiro Iwaya, Akiko Suzuki, Junichi Iwata

**Affiliations:** 1 Department of Orthodontics and Pediatric Dentistry, School of Dentistry, University of Michigan, Ann Arbor, MI 48109, USA; 2 Department of Diagnostic & Biomedical Sciences, School of Dentistry, The University of Texas Health Science Center at Houston, Houston, TX 77054, USA

**Keywords:** craniofacial development, microRNAs, midline facial cleft, cranial neural crest cell

## Abstract

Midline facial clefts are severe craniofacial defects that occur due to an underdeveloped frontonasal process. While genetic studies in mice have identified several genes that are crucial for midfacial development, the interactions and regulatory mechanisms of these genes during development remain unclear. In this study, we conducted a systematic review and database search to curate genes associated with midline facial clefts in mice. We identified a total of 78 relevant genes, which included 69 single-gene mutant mice, nine spontaneous models, and 20 compound mutant mice. We then performed bioinformatic analyses with these genes to identify candidate microRNAs (miRNAs) that may regulate the expression of genes related to midline facial clefts. Furthermore, we experimentally evaluated the four highest-ranking candidates—miR-320-3p, miR-381-3p, miR-27a-3p, and miR-124-3p—in O9-1 cells. Our results indicated that overexpression of any of these miRNAs inhibited cell proliferation through the suppression of genes associated with midline facial clefts. Thus, our results suggest that miR-320-3p, miR-381-3p, miR-27a-3p, and miR-124-3p are involved in the cause of midline facial anomalies.

## 1. Introduction

The midfacial region develops from a pair of maxillary processes and a frontonasal process, which are formed from cranial neural crest (CNC)-derived mesenchyme and surface ectoderm. This process begins in the fourth week of gestation in humans and on embryonic day 9.5 (E9.5) in mice. By the fifth week of gestation in humans and E10.0 in mice, the tip of the frontonasal process thickens, leading to the formation of bilateral nasal placodes. These placodes develop into the lateral and medial nasal processes (LNPs and MNPs), which eventually fuse with the maxillary processes. The merging of the distal ends of the two MNPs raises the intermaxillary segment, which ultimately gives rise to the primary palate that supports the upper incisors by the seventh week of gestation in humans and E12.5 in mice. The overall formation of the midfacial region is completed by the disappearance of the epithelial seams between the maxillary, LNP, and MNP by the seventh week of gestation in humans and E13.5 in mice [1,2]. A notable difference between humans and mice is the presence of a philtrum in humans, whereas mice lack this feature. In humans, the MNPs fuse at the center of the face and extend toward the maxillary processes, resulting in the formation of the philtrum. In contrast, in mice, the fused MNPs are located behind the maxillary processes, which then fuse with each other. While these anatomical differences are apparent, hypoplasia of the frontonasal process and MNP contribute to midline facial clefts in both species. Therefore, mouse models are useful for analyzing the mechanism of midline facial clefts.

Orofacial cleft is classified according to Tessier’s classification [3], which includes the following categories: cleft upper lip only (CLO), cleft lip with cleft palate (CLP), cleft palate only (CPO), midline cleft of the upper lip (also known as median cleft), midline cleft of the lower lip, mandibular cleft, transverse cleft, and oblique cleft. Midline facial cleft occurs due to the underdevelopment of the frontonasal process, which fails to fill the gap between the maxillary processes. The incidence of midline facial clefts is approximately 0.43–0.81% among all upper lip cleft cases, translating to about 1 in 1,000,000 live births [4]. Recent advancements in gene modification techniques have uncovered specific gene expression patterns and single-cell movement during normal craniofacial development, as well as the pathogenic mechanisms involved in craniofacial anomalies. However, the mechanisms underlying midline facial clefts remain unexplored. Like other orofacial clefts, the causes of midline clefts likely involve both genetic and non-genetic factors. Over the past decade, there has been increasing evidence suggesting that environmental factors and epigenetic modifications contribute to the development of clefts and normal growth [5,6,7]. For instance, factors such as non-coding RNAs—including microRNAs (miRNAs) [8], long non-coding RNAs [9], and circular RNAs [10]—as well as DNA methylation [11] and acetylation [12], have been implicated in this context. miRNAs are short single-stranded non-coding RNAs that can inhibit gene expression post-transcriptionally by binding to the 3′ UTR of target mRNAs [13]. The unique aspect of miRNAs is their ability to target multiple genes, while multiple miRNAs can also inhibit a single gene. This characteristic positions miRNA as a crucial modifier of the pathogenesis and prognosis of numerous diseases and physiological conditions. For instance, the contributions of miRNAs to orofacial clefts have been implicated in humans and mice [5,14,15]. In this study, we investigated genes related to midline clefts and their upstream miRNAs to elucidate the miRNA–gene networks linked to the pathogenesis of midline facial clefts in mice, which could potentially lead to new interventions for this condition.

## 2. Results

### 2.1. Collection of a Set of Genes Related to Midline Facial Clefts in Mice

**Figure 1:**
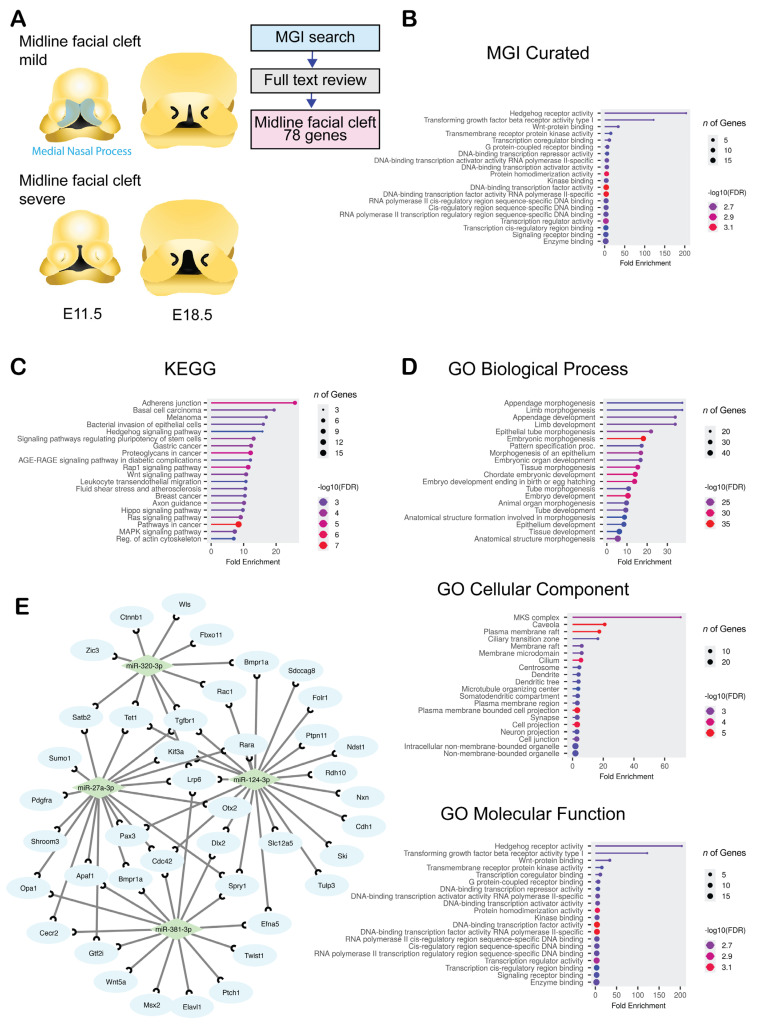
Bioinformatic characterization for genes and miRNAs related to midline facial cleft. (**A**) Drawings of the developing MNP in mice at E11.5 and E18.5. The type of malformation is shown. Blue area shows medial nasal process region. (**B**–**D**) Lollipop graphs for MGI MP (**B**), KEGG (**C**), and GO (**D**) analysis for biological process (BP), cell component (CC), and molecular function (MF). Circle size indicates the number of genes involved. Color code represents −log10 False Discovery Rate (FDR): low (blue) to high (red). (**E**) Visualization of genes related to midline facial clefts and predicted microRNAs. Diamonds (light green) represent predicted miRNAs. Blue circles represent genes related to midline facial clefts.

To gather information on genes related to midline facial clefts, we conducted a thorough search in the Mouse Genomic Informatics (MGI) database, a key resource for mouse genetic and genomic data. Our search identified 78 genes linked to midline facial clefts, derived from 69 single-gene mutations, nine spontaneous mutations, 18 multiple-gene mutations, and two instances related to vitamin A deficiency (Figure 1A and Supplementary Table S1). We performed an MGI grouping analysis, which categorized the genes into relevant groups (Figure 1B and Supplementary Table S2). The identified categories included cleft formation, embryonic development, craniofacial structures, and forebrain development. To further classify these genes by function and associated pathways, we conducted the Kyoto Encyclopedia of Genes and Genomics (KEGG) pathway analysis (Figure 1C and Supplementary Table S3) and Gene Ontology (GO) analysis (Figure 1D and Supplementary Table S4). The KEGG analysis revealed that terms related to cancer, Hedgehog (HH) signaling, and adhesion junctions were significantly enriched. In the GO analysis, we found that genes were considerably enriched in areas concerning limb and appendage development, embryonic morphogenesis, growth factor signaling, primary cilia, and plasma membrane functions. Notably, growth factor-associated genes and transcription factors were markedly enriched across all clusters in both KEGG and GO analyses. This enrichment is logical, given the critical roles of cell growth, proliferation, and survival during embryonic development. HH signaling and its regulatory cellular component, the primary cilium, are essential for craniofacial morphogenesis [16,17]. Genes related to HH signaling and primary cilia clustered prominently in the enriched KEGG and GO analyses.

**Figure 2:**
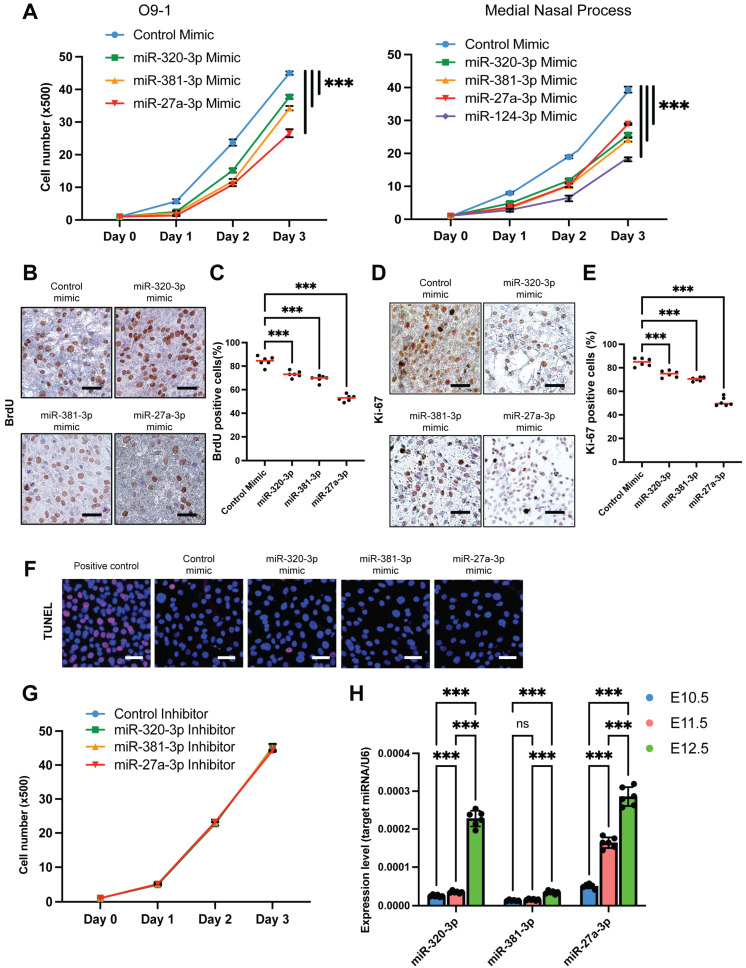
Identification of miRNA functions in the regulation of cell proliferation in O9-1 cells. (**A**) Cell proliferation assays conducted on O9-1 cells (left) and primary mesenchymal cells derived from the MNP (right), treated with the indicated miRNA mimic. *** *p* < 0.001. (**B**) BrdU assays performed on O9-1 cells treated with the indicated miRNA mimic. The scale bar represents 50 μm. (**C**) Quantification of the BrdU assays. *** *p* < 0.001; *n* = 6 per group. Red lines indicate the median. (**D**) Ki-67 immunocytochemical analysis in O9-1 cells treated with the indicated miRNA mimic. Scale bars are 50 μm. (**E**) Quantification of the immunocytochemical analysis for Ki-67. *** *p* < 0.001; *n* = 6 per group. Red lines indicate the median. (**F**) TUNEL assays performed on O9-1 cells treated with the indicated miRNA mimic or a positive control. DAPI was used for nuclear staining. Alexa fluor was used for EdUTP (alkyne-modified dUTP). Scale bars are 50 μm. (**G**) Cell proliferation assays in O9-1 cells treated with the indicated miRNA inhibitor. (**H**) Relative expression levels of the indicated miRNAs in the developing MNP of C57BL/6J mice at embryonic days E10.5 (blue), E11.5 (red), and E12.5 (green). *n* = 6 per group. ns, not significant. *** *p* < 0.001.

**Figure 3:**
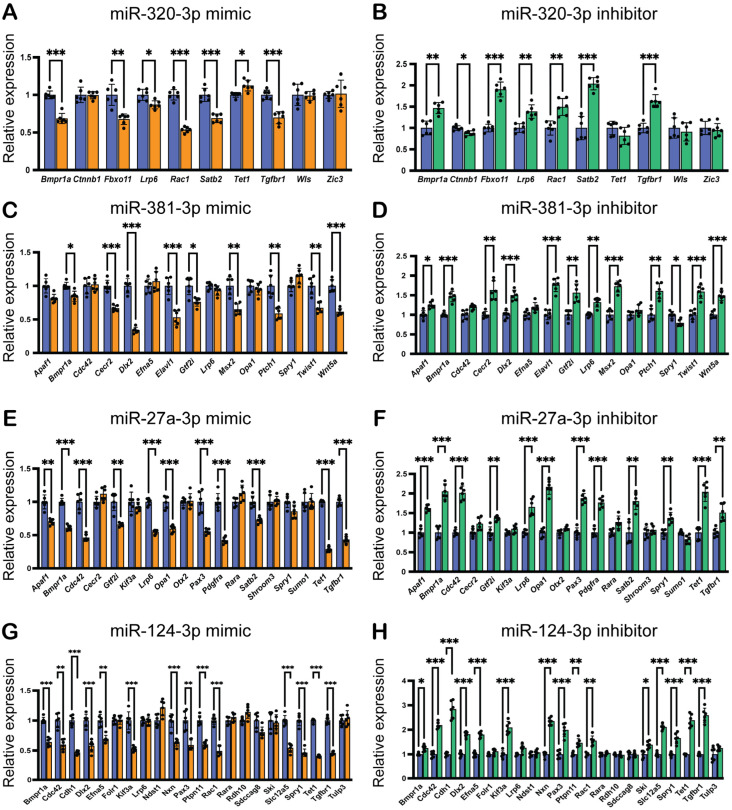
Effects of miRNA mimics and inhibitors on predicted target gene expression in O9-1 cells. (**A**,**C**,**E**,**G**) Quantitative RT-PCR results for target genes in O9-1 cells treated with mimics of either miR-320-3p, miR-381-3p, miR-27a-3p, or miR-124-3p (orange bars) or control (blue bars) for 24 h. * *p* < 0.05, ** *p* < 0.01, *** *p* < 0.001. (**B**,**D**,**F**,**H**) Quantitative RT-PCR results for target genes in O9-1 cells treated with inhibitors of miR-320-3p, miR-381-3p, miR-27a-3p, or miR-124-3p (green bars) or control (blue bars) for the same duration of 24 h, with the same statistical significance markers applied. * *p* < 0.05, ** *p* < 0.01, *** *p* < 0.001.

Next, we examined the regulation of miRNA–target gene interactions using bioinformatic tools. We identified nine candidate miRNAs using miRTarbase, Miranda, PITA, and TargetScan, which are predicted to regulate the expression of genes associated with midline facial clefts (Figure 1E and Table 1). The development of the frontonasal process is influenced by a variety of factors, including CNC cell migration defects, suppressed cell proliferation, induced cell death, or epithelial fusion defects occurring during the early stages of development (between E9.5 and E11.5). As shown in Table 1, most target genes for each miRNA were expressed either in CNC-derived mesenchymal cells and epithelial cells or in CNC-derived cells only. Therefore, in this study, we focused primarily on the effects of miRNAs on CNC-derived mesenchymal cells. We investigated the biological significance of the top four miRNA candidates, which have a *q*-value < 1 × 10^−1^ in the Bonferroni and more than 10 hit counts in the query list (miR-320-3p, miR-381-3p, miR-27a-3p, and miR-124-3p), using O9-1 cells, a mouse embryonic CNC-derived mesenchymal cell line [18], and primary MNP-derived mesenchymal cells (Figure 2 and Figure 3).

### 2.2. Mimics for miR-320-3p, miR-381-3p, and miR-27a-3p Inhibits Cell Proliferation by Suppressing Genes Associated with Midline Facial Clefts in O9-1 Cells and MNP-Derived Mesenchymal Cells

To investigate the role of these candidate miRNAs (miR-320-3p, miR-381-3p, miR-27a-3p, and miR-124-3p) in regulating cell proliferation, we conducted cell proliferation assays using specific mimics for each miRNA in O9-1 cells and primary MNP-derived mesenchymal cells. Our results showed that the overexpression of miR-320-3p, miR-381-3p, and miR-27a-3p significantly inhibited cell proliferation in both cell types (Figure 2A). We further confirmed these results with BrdU incorporation assays (Figure 2B,C) and immunocytochemical analyses for Ki-67 (Figure 2D,E). TUNEL assay for cell death did not show any induction of apoptosis (Figure 2F), indicating that the primary effect of these miRNAs on O9-1 cells is the suppression of cell proliferation. The use of specific inhibitors for these miRNAs did not affect cell proliferation (Figure 2G). Notably, expression of these miRNAs at E10.5 and E11.5 was low in the developing midfacial region and elevated at E12.5 when the development of the midfacial region was completed (Figure 2H). Therefore, we conclude that the overexpression of these miRNAs, rather than their inhibition, leads to decreased cell proliferation in O9-1 cells. Our previous study shows that treatment with miR-124-3p mimic on O9-1 cells and primary MNP-derived mesenchymal cells suppresses cell proliferation, whereas treatment with the miR-124-3p inhibitor does not [19], as observed with the other three candidate miRNAs.

Next, we aimed to validate the predicted miRNA–gene regulation for each miRNA. We performed qRT-PCR analysis on genes in O9-1 cells treated with specific mimics for each miRNA. Our findings revealed that the miR-320-3p mimic significantly downregulated the expression of *Bmpr1a*, *Fbxo11*, *Lrp6*, *Rac1*, *Satb2*, *Tet1*, and *Tgfbr1* in O9-1 cells (Figure 3A). Conversely, the miR-320-3p inhibitor significantly upregulated the expression of the same genes (Figure 3B), indicating that miR-320-3p regulates their expression in a dose-dependent manner. Similarly, the miR-381-3p mimic significantly downregulated the expression of *Bmpr1a*, *Cecr2*, *Dlx2*, *Elavl1*, *Gtf2i*, *Msx2*, *Ptch1*, *Twist1*, and *Wnt5a* in O9-1 cells (Figure 3C), while its inhibitor significantly upregulated the expression of these genes (Figure 3D). The miR-27a-3p mimic significantly downregulated the expression of *Apaf1*, *Bmpr1a*, *Cdc42*, *Gtf2i*, *Lrp6*, *Opa1*, *Pax3*, *Pdgfra*, *Satb2*, *Tet1*, and *Tgfbr1* in O9-1 cells (Figure 3E). Its inhibitor also significantly upregulated the expression of these genes (Figure 3F). Our previous study shows that miR-124-3p mimic can inhibit cell proliferation in O9-1 cells [20]. We found that miR-124-3p mimic significantly downregulated the expression of midline facial cleft-related genes (*Bmpr1a*, *Cdc42*, *Cdh1*, *Dlx2*, *Efna5*, *Kif3a*, *Nxn*, *Pax3*, *Ptpn11*, *Rac1*, *Slc12a5*, *Spry1*, *Tet1*, and *Tgfbr1*) in O9-1 cells (Figure 3G), while its inhibitor upregulated the expression of *Bmpr1a*, *Cdc42*, *Cdh1*, *Dlx2*, *Efna5*, *Kif3a*, *Nxn*, *Pax3*, *Ptpn11*, *Rac1*, *Ski*, *Slc12a5*, *Spry1*, *Tet1*, and *Tgfbr1* (Figure 3H). Thus, expression of most of the candidate miR-target genes was regulated in an miR dose-dependent manner. Interestingly, among these candidate genes, *Bmpr1a* was commonly suppressed in all miRNA mimics.

## 3. Discussion

Midline facial cleft results from a failure in the growth and fusion of frontonasal and maxillary processes during the early stages of craniofacial development. Recent studies have uncovered the important roles of specific microRNAs (miRNAs)—such as miR-124-3p, miR-149, and miR-470-5p—in the development of cleft lip in mice [21,22,23]. Notably, this study expands our understanding by identifying potential causative miRNAs for midline facial cleft, including miR-186-5p, miR-320-3p, miR-381-3p, and miR-27a-3p, which are distinct from those associated with cleft lip [5,24]. Currently, the role of miR-320-3p, miR-381-3p, and miR-27a-3p in developmental and disease contexts remains largely unknown. miR-186-5p has been investigated in several cancers and diseases [25,26,27,28,29,30]. For instance, miR-186-5p inhibits oral squamous cell carcinoma progression by targeting the integrin subunit alpha 6 (ITGA6) gene, thus affecting the phosphoinositide 3-kinase (PI3K)/AKT signaling pathway [31].

The intricate processes of craniofacial development involve coordinated growth and rotation of facial primordia, which are regulated by specific genes [32,33]. This suggests that there is a need for distinct mechanisms tailored to each facial primordium’s development. For instance, although severe bilateral cleft lip and midline facial cleft present similar outward appearances, their mechanisms may differ in each cleft type [34,35]. Our study shows that miRNAs associated with each cleft type differ, indicating that each facial primordium is regulated by its specific set of miRNAs, which operate in a spatiotemporal manner. It is worth highlighting that a significant majority—48 of 69 genes and 7 of 9 spontaneous mouse lines related to midline facial clefts—are unique to this condition. Mutations in these genes often also lead to complications such as exencephaly or neural tube defects, suggesting a complementary relationship between frontonasal and forebrain development. Furthermore, miR-124-3p stands out as a common factor across various types of clefts, including cleft lip, cleft palate, and midline cleft in mice [23,36], as well as cleft lip in humans [37]. This emphasizes miR-124-3p’s potential as a critical pathogenic miRNA involved in craniofacial malformations and as an influential epigenetic modifier in the development of facial clefts.

The treatment with miRNA mimics significantly reduced cell proliferation; however, the miRNA inhibitor did not have any effect on cell proliferation in O9-1 cells and primary mesenchymal cells derived from MNPs. The expression levels of the candidate miRNAs remained very low in both O9-1 cells and primary MNP-derived mesenchymal cells under physiological conditions. These low levels may be enough to induce gene expression and enhance the effects on cell proliferation. As a result, the inhibitor did not have a functional impact on cell proliferation. Predicting miRNA involves determining whether a gene contains a specific miRNA-binding sequence in its 3′ untranslated region (3′ UTR). This prediction is influenced by various factors, including cell lineage, the type of tissue or organ, specific conditions (such as during development, homeostasis, or pathological states), and the spatiotemporal expression patterns of the gene. It is well established that gene expression undergoes significant changes during embryonic development, with variations occurring in a short time frame across different locations and cell lineages. Therefore, the target genes predicted by miRNAs are not expressed continuously or ubiquitously. In addition, some miRNA-binding sequences on target genes may not perfectly match the ideal sequence. This mismatch can affect the efficiency of gene regulation, similar to the role of enhancers found in the 5′ UTR. For genes that do not respond to treatment with miRNA mimics or inhibitors, possible reasons include that these genes are not expressed in CNC-derived O9-1 cells, the miRNA-binding site is non-functional, or a combination of activities from multiple miRNAs is necessary for regulation. These findings are crucial for understanding the complex mechanisms of miRNA regulation and their potential implications in craniofacial development.

While this study has made important strides, there are limitations regarding the functional validation of the candidate causative miRNAs. For instance, a direct binding assay between miRNAs and their target genes would provide strong in vitro validation for the regulation of these genes by the identified miRNAs. In addition, due to limitations regarding the availability of ectodermal cells from the MNP, this study primarily focused on mesenchymal cells derived from CNC cells. We incorporated information on the expression patterns of target genes from the existing literature and confirmed that most genes are expressed either ubiquitously or specifically within CNC-derived mesenchyme. Some genes associated with midline facial clefts were found to be expressed in both mesenchyme and epithelium. We excluded epithelial-specific genes from our list of candidate genes in mesenchymal cells. For example, *Shroom3* and *Sumo1* (associated with miR-27a-3p) and *Folr1* (associated with miR-124-3p) were specifically expressed in epithelial cells. Our findings indicate that the expression of these genes in mesenchymal cells was not influenced by treatment with either a specific miRNA mimic or inhibitor. Therefore, we plan to evaluate the expression of these genes in ectodermal cells in a future study. Another limitation of this research is the lack of in vivo validation. Mice with genetically manipulated miRNA expression would be invaluable for investigating midline facial development. To build upon our findings, future research should focus on conducting additional studies in cultured epithelial cells and in vivo settings to validate the roles of these miRNAs in midline facial clefts. The significance of this validation is profound, and it could greatly deepen our understanding of craniofacial development and potentially lead to innovative treatments for facial clefts.

In summary, we have identified 24 genes related to midline facial clefts based on craniofacial phenotypes observed in genetically engineered mice. By examining these genes, we pinpointed and characterized three candidate miRNAs that are crucial for cell proliferation and target gene regulation.

## 4. Materials and Methods

### 4.1. Gene Search for Mouse Midline Facial Clefts and Bioinformatics Analysis

A comprehensive gene search for midline facial clefts in mice was conducted using the MGI database (https://www.informatics.jax.org) in September 2024, with terms related to facial clefts and cleft lip. Each gene was evaluated alongside relevant literature for mouse phenotypes, and only those exhibiting a midline cleft phenotype were included in the analysis. To predict miRNA–target gene regulation, several tools, including miRTarbase, miRanda, PITA, and TargetScan, were utilized, as previously described [23]. Fisher’s exact test was employed to assess the significance of shared genes among miRNA targets and genes associated with midline facial clefts in mice. To ensure the robustness of our findings, the Benjamini–Hochberg procedure was applied for multiple test corrections. Pathway analysis was conducted using the Kyoto KEGG and GO enrichment analysis (http://www.geneontology.org) was performed using ShinyGO 0.81 [38], focusing on biological process (BP), cellular component (CC), and molecular function (MF) [20,21,38,39]. Categories with significant enrichment for the relevant genes were filtered using a false discovery rate (FDR)-adjusted *p*-value of less than 0.05, requiring at least four genes associated with midline facial clefts. The *p*-value was calculated through a hypergeometric test, and to avoid overly general GO terms, we applied a hierarchical level 4 cut-off.

### 4.2. Cell Culture

The O9-1 cells [18], a mouse cranial neural crest cell line (SCC049, Millipore Sigma, Burlington, MA, USA), were cultured, as previously described [6].

### 4.3. Isolation of Primary Medial Nasal Process Mesenchymal Cell

The animal protocol (PRO00011979) was approved by the Animal Welfare Committee and the Institutional Animal Care and Use Committee at the University of Michigan. Primary MNP-derived cells were isolated from the MNP of E11.5 C57BL/6J mice (strain #000664, The Jackson Laboratory, Bar Harbor, ME, USA). We pooled tissues from three embryos and treated them as one sample. We dissected the MNP and created a single-cell suspension using 0.25% trypsin and 0.05% EDTA for 10 min at 37 °C in an atmosphere with 5% CO_2_. We maintained primary MNP-derived mesenchymal cells in Advanced DMEM (ThermoFisher Scientific, Waltham, MA, USA) supplemented with 10% fetal bovine serum, penicillin and streptomycin, β-mercaptoethanol, MEM non-essential amino acids, L-glutamine, and sodium pyruvate at 37 °C in a humid environment with 5% CO_2_.

### 4.4. Cell Proliferation and Cell Death Assay

O9-1 cells or primary MNP-derived mesenchymal cells were plated in 96-well cell culture plates at a density of 500 cells (O9-1) or 5000 cells (MNP) per well. The cells were treated with a mimic for negative control, miR-320-3p, miR-381-3p, and miR-27a-3p, as well as miR-124-3p or an inhibitor for negative control, miR-320-3p, miR-381-3p, and miR-27a-3p, as previously described [6]. Cell proliferation was assessed using the Cell Counting Kit 8 (Dojindo Molecular Technologies, Inc., Rockville, MD, USA) at 24, 48, or 72 h after treatment (*n* = 6 per group), as previously described [6]. Cell death was analyzed using the TUNEL assay (Click-iTTM Plus TUNEL assay kit; ThermoFisher Scientific) following the manufacturer’s instructions (*n* = 3 from 3 independent experiments, 6 technical replicates).

### 4.5. Quantitative RT-PCR

O9-1 cells were treated at 80% confluence with either a mimic or an inhibitor for miR-320-3p, miR-381-3p, miR-27a-3p, or miR-124-3p, as well as a negative control, following previously described methods [6]. The PCR primers used in this study are detailed in Supplementary Table S6. The expression of each gene was normalized to *Gapdh* expression. miRNA expression was measured, following previously described methods [6]. *n* = 6 from 6 independent experiments.

### 4.6. Statistical Analysis

A two-tailed non-parametric Student’s t-test was utilized for comparisons between two groups. For multiple comparisons, one-way analysis of variance (ANOVA) along with Bonferroni correction or the Tukey–Kramer post hoc test was applied. The cell proliferation assays were analyzed using two-way ANOVA. Cell proliferation and cell death assays were derived from 3 independent experiments (6 technical replicates). RT-PCR results were derived from 6 independent experiments. All experimental analyses were performed using Prism software (GraphPad Software Ver.10.6.1, Boston, MA, USA). A *p*-value of less than 0.05 was considered statistically significant. The data are presented as mean ± standard deviation (SD) in all graphs.

## Figures and Tables

**Table 1:** miRNAs predicted from target genes.

Sequence	miRNA Family	*q*-Value Bonferroni	*q*-Value FDR B&H	Hit Count in Query List	Target Gene
AAAGCUG	miR-320-3p	9.45 × 10^−5^	1.42 × 10^−2^	10	*Bmpr1a*, *Ctnnb1*, *Fbxo11*, *Lrp6*, *Rac1*, *Satb2*, *Tet*1, *Tgfbr1*, *Wls*, *Zic3*
AUACAAG	miR-381-3p	1.32 × 10^−6^	4.40 × 10^−8^	15	*Apaf1*, *Bmpr1a, Cdc42*, *Cecr2*, *Dlx2*, *Efna5*, *Elavl1*, *Gtf2i*, *Lrp6*, *Msx2*, *Opa1*, *Ptch1*, *Spry1*, *Twist1*, *Wnt5a*
UCACAGU	miR-27a-3p	1.99 × 10^−2^	6.90 × 10^−5^	18	*Apaf1*, *Bmpr1a*, *Cdc42*, *Cecr2*, *Gtf2i*, *Kif3a*, *Lrp6*, *Opa1*, *Otx2*, *Pax3*, *Pdgfra*, *Rara*, *Satb2*, (*Shroom3*), *Spry1*, *Sumo1*, *Tet1*, *Tgfbr1*
AAGGCAC	miR-124-3p	9.98 × 10^−4^	8.25 × 10^−6^	22	*Bmpr1a*, *Cdc42*, *Cdh1*, *Dlx2*, *Efna5*, *Folr1*, *Kif3a*, *Lrp6*, *Ndst1*, *Nxn*, *Pax3*, *Ptpn11*, *Rac1*, *Rara*, *Rdh10*, *Sdccag8*, *Ski*, *Slc12a5*, *Spry1*, *Tet1*, *Tgfbr1*, *Tulp3*
ACAGUAU	miR-144-3p	6.23 × 10^−1^	8.10 × 10^−3^	5	*Bmpr1a*, *Efna5*, *Pax3*, *Ptch1*, *Rac1*, *Tet1*, *Tgfbr1*
ACAUUAC	miR-323-3p	3.54 × 10^−1^	3.53 × 10^−2^	3	*Chd1*, *Fgfr1*, *Gldc*, *Ndst1*, *Ski*, *Sumo1*, *Wnt5a*
ACCACAG	miR-140-3p	2.16 × 10^−1^	3.27 × 10^−2^	3	*Ndst1*, *Nxn*, *Otx2*, *Ptch1*, *Satb2*, *Wnt5a*
AGCUGCC	miR-22-3p	2.49 × 10^−1^	1.87 × 10^−2^	3	*Cecr2*, *Elavlq*, *Fgfr1*, *Satb2*, *Ski*, *Tet1*, *Tgfbr1*
AUAAAGU	miR-142a-5p	6.37 × 10^−1^	4.43 × 10^−3^	5	*Ctnnb1*, *Efna5*, *Otx2*, *Pdgfra*, *Snx3*, *Tacc3*, *Tfap2A*, *Tulp3*

B&H: The Benjamini–Hochberg Procedure. Underlined genes are expressed only in CNC-derived mesenchymal cells. Double-underlined genes are expressed only in epithelial cells. A gene in parentheses is not expressed in developing frontonasal and maxillary processes.

## Data Availability

The original contributions presented in this study are included in the article and Supplemental Materials. Further inquiries can be directed to the corresponding author.

## Supplementary Materials

The following supporting information can be downloaded at https://www.mdpi.com/article/10.3390/ijms262110730/s1.
