# Supplementary material for: Overexpression of miR-320-3p, miR-381-3p, and miR-27a-3p Suppresses Genes Related to Midline Facial Cleft in Mouse Cranial Neural Crest Cells
Source: Int J Mol Sci. 2025 Nov 4;26(21):10730. doi: 10.3390/ijms262110730 (PMC12609918; doi:10.3390/ijms262110730)
Supplement: Supplementary file 1 [file ijms-26-10730-s001.zip › Table S6 (midline facial cleft).pdf]

Table S6. Primers used in this study

Gene	Forward Sequence	Reverse Sequence
Apaf1	CCCCAGAGGCCGGTTATTTT	CAGAGACCTTGGGTGTTTGC
Bmpr1a	CCCCTGTTGTTATAGGTCCGT	TTCACCACGCCATTTACCCA
Cdc42	GATTGGTGGAGAGCCATACACTC	TGAGGATGGAGAGACCACTGAG
Cdh1	GGTCATCAGTGTGCTCACCTCT	GCTGTTGTGCTCAAGCCTTCAC
Cecr2	AGGAAACAGCCACCAGTGGAGA	GAGAGCCAAATGTTCCTGGTGC
Ctnnb1	AAGGTAGAGTGATGAAAGTTGTT	CACCATGTCCTCTGTCTATTC
Dlx2	TGGCTGATATGCACTCGACC	TGCTTTTGGCGGAGTAGGAG
Efna5	TACGCCGTCTACTGGAACAGCA	GTCTTCTGGGACAGAGTCCTCA
Elavl1	ACTGAACGGCTTGAGACTCCAG	CCACATCCTTCTGTGTCATGGTC
Fbxo11	TCAAGCAGGTGTCCTCATCAGC	CTGATTGCCTTCGAGTGTTGCAG
Folr1	GGACTGAACTTCTCAATGTCTGC	CTTCCTGGCTTGTGTTCGTGGA
Gtf2i	CCTCGTGGTATGGAATCCCAAG	GTGTTTGTCCGTGGCTGAGTCA
Kif3a	TGCCGTATCGGAACTCCAAA	CTTTCTTTTACCTGCTTGGTCCC
Lrp6	ACTTGCCTCATCCTTTTGGC	TGTTGGCACGTTCAATGCTG
Msx2	AAGACGGAGCACCGTGGATACA	CGGTTGGTCTTGTGTTTCCTCAG
Ndst1	GCGAACAGAACCTGCCAAAGTC	CTCCTTCAAGCAGTTGGCACCA
Nxn	CTCGTCTCAACCGACTGTATGG	AGTCCTCGTCATTGAGCACCTC
Opa1	TCTCAGCCTTGCTGTGTCAGAC	TTCCGTCTCTAGGTTAAAGCGCG
Otx2	TGAGGGAAGAGGTGGCACTGAA	GCCTCACTTTGTTCTGACCTCC
Pax3	TACAGACAGCTTTGTGCCTCC	AGATAATGAAAGGCACTTTGTCCAT
Pdgfra	TCAGAGAGAATCGGCCCCA	AGGACGAATTCAGCTGCACA
Ptch1	TAGCCCTGTGGTTCTTGTCC	TGTGGTCATCCTGATTGCAT
Ptpn11	TGACTTCTGGCGGATGGTGTTC	GACGTTCCTAACACGCATGACC
Rac1	TTGGTAAAACCTGCCTGCTCA	AAGAACACGTCTGTCTGCGG
Rara	AGCACCAGCTTCCAGTCAG	AGCAAGGCTTGTAGATGCGG
Rdh10	TGGTCAACTGCCACGCACACTT	CCTCAACTCCAGCAGTGCTGAA
Satb2	CCTCAAAATCACACACCAGCA	GGGACCTTGGTGTTGGAACTA
Sdccag8	CAGACCATCGAGAGGCTGACTA	CACCTGCTCATAGGCACTTGTC
Shroom3	TGGCACAGATGACTTTCCTCCG	CTTCCCTTGTGACCATCACCCT
Ski	GCGTCTTCTGAGAAGGACAAGC	CTTTCTCACTCGCAGACACTGC
Slc12a5	CTCCTCAATGCCACCTGTGATG	GGTCAGGTAAGAACTCCACAGG
Spry1	CACTAGCCGGCGTGGC	TCTCCAGTTCCAGCAGTCAG
Tet1	TGCCAGCAGAAGGCCAACT	TCTTTTCCCTCTGGGGCCT
Tgfbr1	TGCTCCAAACCACAGAGTAGGC	CCCAGAACACTAAGCCCATTGC
Tulp3	CCAGGGATAAGAAAGGCATGGAC	GTCTGTGGAGACCAGGTAGTTG
Twist1	GATTCAGACCCTCAAACTGGCG	AGACGGAGAAGGCGTAGCTGAG
Wls	TTGCTGTTGGCTCCTTCTGCCT	GGCAGATACCTGCCACAATGATG
Wnt5a	GGAACGAATCCACGCTAAGGGT	AGCACGTCTTGAGGCTACAGGA
Zic3	CACACTGGCGAGAAACCCTTCC	GTTGGCAAACCGTCTGTCACAG
