# Supplementary material for: Overexpression of miR-320-3p, miR-381-3p, and miR-27a-3p Suppresses Genes Related to Midline Facial Cleft in Mouse Cranial Neural Crest Cells
Source: Int J Mol Sci. 2025 Nov 4;26(21):10730. doi: 10.3390/ijms262110730 (PMC12609918; doi:10.3390/ijms262110730)
Supplement: Supplementary file 1 [file ijms-26-10730-s001.zip › Table S1 (midline facial cleft).pdf]

Supplementary Table S1: Genes related to midline facial cleft

#	Gene symbol	Chr	Description	Mouse genotype	Reference (PMID)	cell lineage expression in craniofacial region
**Single gene mutant mice**						
1	*Apaf1*	10	apoptotic peptidase activating factor 1	Global-ENU-induced-point mutation and gene trapped. Homozygous	9216040; 10656767; 16887819	ubiquitous
2	*B9d1*	11	B9 protein domain 1	Conditional KO (KI-LacZ) (*B9d1*$^{LacZ/LacZ}$)	21677750 (global KO project. No detail information is available); 21763481	Expressed in ciliated cells
3	*Bmpr1a*	14	bone morphogenetic protein receptor, type 1A	*Mpz-Cre;dnBmpr1a*$^{F/F}$	22773757	ubiquitous
4	*Cdc42*	4	cell division cycle 42	*Wnt1-Cre;Cdc42*$^{F/F}$	24056078	ubiquitous
5	*Cecr2*	6	CECR2, histone acetyl-lysine reader	Global KO. Homozygous	20589882	ubiquitous
6	*Cdh1 (aka E-cad)*	8	cadherin 1	*Wnt1-Cre2;Cdh1*$^{F/F}$	37225711	both epithelial and CNC-derived mesenchymal cells (migrating CNC cells) (26879760; 26235046)
7	*Ctnnb1*	9	catenin (cadherin associated protein), beta 1	*Wnt1-Cre;Ctnnb1*$^{F/F}$, *CAG-Cre;Ctnnb1*$^{Y654E/Y654E}$, *Ctnnb1*$^{E654/E654}$, *Foxg1*$^{Cre/+}$*:Ctnnb1*$^{F/F}$	11262227; 21307168; 21571225	ubiquitous
				Pitx1-Cre;Ctnnb1$^{ex2-6/ex2-6}$ (loss of function) and *Pitx1-Cre;Ctnnb1*$^{ex3/ex3}$ (gain of function)	22354888	
8	*Ctnnbip1* (aka Icat)	4	catenin beta interacting protein 1	Global KO	15148409	ubiquitous
9	Dlx2	2	distal-less homeobox 2	*Wnt1-Cre;iZEG-Dlx2* (conditional overexpression of *Dlx2*)	23246068	both epithelial and mesenchymal cells
10	*Edn1* (aka *Et1*)	13	endothelin 1	*Wnt1-Cre;Edn1*^{Tg} (overexpression)	25725491	highly expressed in both epithelium and mesenchyme of the branchial arch
11	*Efna5*	17	ephrin A5	Global KO. Homozygous	9491985 (No image is available, only text)	ubiquitous
12	*Elavl1*	8	ELAV (embryonic lethal, abnormal vision)-like 1 (Hu antigen R)	*Sox2-Cre;Elavl1*$^{F/F}$	19307312	cranial neural crest cells (36189921)
13	*Ext1*	15	exostosin glycosyltransferase 1	*Wnt1-Cre;Ext1*$^{F/F}$	19509472	ubiquitous
14	*Fbxo11* (aka *Gena104*, *Jf*)	17	F-box protein 11	Global-ENU-induced single point mutation and global-ENU-induced hypomorph. Homozygous	17035249; 19580641; 10932191	ubiquitous
15	*Fgf8*	19	fibroblast growth factor 8	Global KO. Homozygous	12223415	Expressed in the epithelium of lateral and medial nasal processes (16267092) and AER epithelium (11101845)
				Nes-Cre;Fgf8$^{F/null}$	10601039	
16	*Fgfr1* (aka *Eask*, *Flt2*, *Hspy*)	8	fibroblast growth factor receptor 1	*Wnt1-Cre;Fgfr1*$^{F/F}$	23754280	CNC-derived mesenchymal cells
17	*Folr1* (aka *Folbp1*)	7	folate receptor 1 (adult)	Global KO. Homozygous	12854656	epithelial cells (25564455)

18	*Foxd3* (aka *Hfh2*)	4	forkhead box D3	*Wnt1-Cre;Foxd3*$^{F/-}$ and *Wnt1-Cre;Foxd3*$^{F/F}$	18367558	ubiquitous, especially CNC derived cell (18367558)
				Pax3-Cre$^{/+}$*;Foxd3*$^{F/-}$	21254333	
19	*Gldc*	19	glycine decarboxylase	Global-ENU-induced single point mutation. Homozygous	25807483 (global KO project. No information is available in the MS, only MGI without image)	ubiquitous
20	*Grhl2*	15	grainyhead like transcription factor 2	Global-ENU-induced (*Grhl2*$^{Clft3/Clft3}$), Global-KKI-LacZ (*Grhl2*$^{LacZ/LacZ}$), and Global-gene tapped. Homozygous	21515572; 26177923; 21377456: 20654612 (no image is available, only text)	epithelial cells (32005677)
				Crisp/Cas9 (*Grhl2*$^{m1Nisw/m1Nisw}$)		
21	*Gtf2i*	5	general transcription factor II I	Global KO. Homozygous	19109438	ubiquitous
22	*Gtf2ird1*	5	general transcription factor II I repeat domain-containing 1	Global KO. Homozygous	19109438	epithelial cells, odontoblast,s and tongue (17239664)
23	*Hand1*	11	heart and neural crest derivatives expressed 1	*Wnt1-Cre2;Hand1*$^{PO4-/F}$, *Wnt1-Cre2;Hand1*$^{PO4+/F}$, *Hand1*$^{PO4-/+}$, and *Hand1*$^{PO4+/+}$ (all *Hand1* phospho mutant)	25053435	Post-migrated ventral most cap of the pharyngeal arch CNC cells (25053435)
24	*Ift88* (aka *Tg737*)	14	intraflagellar transport 88	*Wnt1-Cre;Ift88*$^{F/F}$	27802276/ 28069795	ubiquitous
25	*Kif3a*	11	kinesin family member 3A	*Wnt1-Cre;Kif3a*$^{F/F}$	27802276/ 20106874/ 21429982 (K14)	ubiquitous
26	*Kif7*	7	kinesin family member 7	Global-ENU-induced single point mutation. Homozygous	25807483 (global KO project. No information is available in MS, only MGI)	ubiquitous
27	*Lrp6* (aka *Ska26*, *Skax26*, *Cd*, *Gw*)	6	low density lipoprotein receptor-related protein 6	Global KO. Homozygous	19700620	ubiquitous
				CMV-Cre;Lrp6$^{floxdel/floxdel}$	19653321	
28	*Mks1*	11	MKS transition zone complex subunit 1	Global-ENU-induced-deletion. Homozygous	21045211; 23454480	ubiquitous
29	*Mmachc*	4	methylmalonic aciduria cblC type, with homocystinuria	Global KO. Homozygous	No MS, only MGI and IMPC	Expressed in both mesenchyme and epithelium (21536470)
30	*Morc2a*	11	microrchidia 2A	Global KO. Homozygous	No MS, only MGI and IMPC	ubiquitous
31	*Msx2*	13	msh homeobox 2	Global-transgenic (*Tg*$^{WT/WT}$ and *Tg*$^{P148H/P148H}$). Homozygous	9147639	Expressed in both epithelium and mesenchyme
32	*Mthfd1l*	10	methylenetetrahydrofolate dehydrogenase (NADP+ dependent) 1-like	Conditional KO (KI-LacZ) (*EIIa-Cre;Mthfd1l*$^{LacZ/LacZ}$). Homozygous	23267094	ubiquitous (24733394)
33	*Ndst1*	18	N-deacetylase/N-sulfotransferase (heparan glucosaminyl) 1	Global KO. Homozygous	16020517; 17183530	ubiquitous
34	*Nosip*	7	nitric oxide synthase interacting protein	Global KO. Homozygous	25546391	CNC-derived cells (not analyzed in the ectoderm during craniofacial development)

35	*Nxn*	11	nucleoredoxin	Global-CRISPR/Cas9-KO and global KO (KI-LacZ). Homozygous	Reference is open data base. No image is available, only in MGI	ubiquitous
36	*Opa1*	16	OPA1, mitochondrial dynamin like GTPase	Global-ENU-induced. Homozygous	20652258	ubiquitous
37	*Otx2*	14	orthodenticle homeobox 2	Global KO. Heterozygous	7590242 (Not mentioned in text, but figures from bone staining are provided)	ubiquitous
38	*Pak1ip1*	13	PAK1 interacting protein 1	Global-ENU-induced point mutation (mray/mray). Homozygous	23935987	ubiquitous (highly expressed in facial primordium and forebrain) (23935987)
39	*Pax3*	1	paired box 3	$Pax3^{pax3\text{-}ERD/GFP}$ (Pax3-ERD: dominant negative of *Pax3*)	25800090	CNC-derived cells (2022185)
40	*Pdgfra*	5	platelet derived growth factor receptor, alpha polypeptide	Global-KO, homozygous and $Wnt1\text{-}Cre;Pdgfra^{F/F}$	9226440; 12490557	CNC-derived mesenchymal cells (1322271; 9510033)
				$Sox10ER^{T2}CreER^{T2};Pdgfra^{F/F}$	26250625	
41	*Piga*	X	phosphatidylinositol glycan anchor biosynthesis, class A	$Wnt1\text{-}Cre;Piga^{F/Y}$ (hemizygous) and $EIIa\text{-}Cre;Piga^{F/F}$	31232685; 10377440	Expressed in the mesenchyme (31232685)
42	*Porcn*	X	porcupine O-acyltransferase	$Wnt1\text{-}Cre;Porcn^{F/Y}$, $Rx3\text{-}Cre;Porcn^{F/Y}$, and $Wnt1\text{-}Cre;Rx3\text{-}Cre;Porcn^{F/Y}$	25451153	CNC-derived mesenchymal cells (21554866)
43	*Ptch1*	13	patched 1	$Wnt1\text{-}Cre;Ptch1^{F/F}$	23900075	both CNC-derived mesenchymal cells and epithelial cells (15107405)
44	*Ptpn11* (aka *Shp2*)	5	protein tyrosine phosphatase, non-receptor type 11	$Wnt1\text{-}Cre;Ptpn11^{Tg(Q79R)}$ (gain-of-function)	19706403	both CNC-derived mesenchymal cells and epithelial cells
				$Wnt1\text{-}Cre;Ptpn11^{F/F}$ (loss-of-function)	19541608	
45	*Rac1*	5	Rac family small GTPase 1	$Wnt1\text{-}Cre;Rac1^{F/F}$	20184871	both CNC-derived mesenchymal cells and epithelial cells (22553120)
46	*Rara*	11	retinoic acid receptor, alpha	Global KI (dominant negative Rara403)	8096643	both CNC-derived mesenchymal cells and epithelial cells (10842077)
47	*Rax* (aka *Ey1*, *Rx*)	18	retina and anterior neural fold homeobox	Global KO ($Rax^{\Delta1\text{-}2/\Delta1\text{-}2}$ and $Rax^{\Delta2/\Delta2}$). Homozygous. Maybe $Rax^{neo/neo}$ (hypomorph or null) too.	9177348; 15789424	prospective forebrain and optic vesicle at E8.5 and only lens after E10.5 (9096350)
48	*Rdh10*	1	retinol dehydrogenase 10 (all-trans)	Global-KO ($Rdh10^{-/-}$) and global-KO-KI-EGFP ($Rdh10^{eGFP/eGFP}$). Homozygous	21930923	restricted expression in each developmental stage. The epithelium and mesenchyme of distal tip of the LNP at E12.5-E14.5. Nasal side epithelium of the palatal shelf, MEE, and the epithelium and mesenchyme of distal tip of the maxilla and mandible, dental papillae at E14.5. (17849458)
49	*Satb2*	1		Global KO (KI-Cre) ($Satb2^{Cre/Cre}$). Homozygous	16960803	The mesenchyme of distal maxillary process and medial nasal process junction and distal mandibular process after E10.5. Not expressed in the midline. At E12.5, palatal mesenchyme, mandible, and
			special AT-rich sequence binding protein 2	Global KO (KI-LacZ) ($Satb2^{LacZ/LacZ}$). Homozygous	16751105	

						tongue mesenchyme. (16960803)
50	*Shroom3*	5	shroom family member 3	Global gene trapped and ENU-induced. Homozygous	10589677	forebrain and skeletal muscle (10589677)
51	*Ski*	4	ski sarcoma viral oncogene homolog (avian)	Global KO. Homozygous	9284043	CNC-derived cells (7894074)
52	*Slc12a5* (aka *KCC2*)	2	solute carrier family 12, member 5	Transgenic (*Slc12a5*^{FL}) (overexpression-full length- under nestin promotor) and transgenic (*Slc12a5*$^{\Delta NTD}$) (N-terminal deletion-under nestin promotor)	20529123	neural crest cells (20529123)
53	*Snx3*	10	sorting nexin 3	Global KO. Homozygous	No MS and images are available. MGI direct data deposition in 2008. Some images are in IMPC.	The neural tube and branchial arch at E9.5 and ubiquitous after E12.5 (20529123)
54	*Sp8* (aka *mBtd*)	12	trans-acting transcription factor 8	Global KO. Homozygous	14597661; 23872235	The anterior neural ridge at E9.5. nasal pit epidermal (inner side of the LNP and MNP) and medial telencephalon at E10.5 (23872235)
				Pax3-Cre;Sp8$^{F/-}$	23872235	
				Foxg1-Cre;Sp8$^{F/-}$		
				Mesp1-Cre;Sp8$^{F/-}$		
55	*Spry1*	3	sprouty RTK signaling antagonist 1	*Wnt1-Cre;Spry1*$^{F/F}$	20459789	Expressed in migrating and post-migratory CNC cells: the branchial arch, the FNP, boundary of mid- and hind-brain at E9.5 (10498682; 20459789)
56	*Sumo1* (aka *Pic1*, *Smt3C*, *Ubl1*)	1	small ubiquitin-like modifier 1	Global-gene trapped-hypomorph-KI-LacZ (*Sumo1*$^{LacZ/+}$). Heterozygous	16990542 (no image is available for midline cleft, only described in text)	The epithelium of upper lip, primary palate, secondary palate, and the MEE at E13.5-E14.5 (16990542)
57	*Tacc3*	5	transforming, acidic coiled-coil containing protein 3	Global KO. Homozygous	11847113 (no image is available. Only described in text)	CNC cells in *Xenopus* (31031646) no MS in mice.
58	*Tbc1d32*	10	TBC1 domain family, member 32	Global-ENU-induced-point mutation. Homozygous	25807483 (global KO project. no information is available in MS, only in MGI. Midline cleft images are in MGI)	unknown
59	*Tfap2a*	13	transcription factor AP-2, alpha	Global KO. Homozygous	8622765	CNC-derived mesenchymal cells and surface ectodermal cells (1989904)
60	*Tgfbr1* (aka *Alk5*)	4	transforming growth factor, beta receptor I	*Wnt1-Cre;Tgfbr1*$^{F/F}$	16806156	ubiquitous
61	*Tmem107*	11	transmembrane protein 107	Global-ENU-induced-point mutation (p.E125G). Homozygous	22698544; 28954202	maybe both mesenchyme and epithelium
62	*Tulp3*	6	tubby-like protein 3	Global KO (KI-GFP) (*Tulp3*$^{GFP/GFP}$). Homozygous	11406614	CNC-derived mesenchymal cells and epithelial cells (11406614)
63	*Twf2*	9	twinfilin actin binding protein 2	Global-ENU-induced-point mutation. Homozygous	25807483 (global KO project. no information is available in MS, only in MGI. Midline cleft images are in MGI)	unknown
64		12	twist basic helix-loop-helix	*HtPA-Cre;Twist1*$^{F/del}$	19414008	CNC-derived mesenchymal cells (31171666; 19414008)

	Twist1 (aka *Ska10*)		transcription factor 1	*Wnt1-Cre;Twist1*$^{F/del}$<br>*Tyr-Cre;Twist1*$^{F/del}$		
65	*Wdpcp*	11	WD repeat containing planar cell polarity effector	Global-ENU-induced-point mutation (c.224A>G, p.S54X). Homozygous	24302887 (no image for CP)	ubiquitous
66	*Wls* (aka *Gpr177)*	3	wntless WNT ligand secretion mediator	*Foxg1-Cre;Wls*$^{F/F}$	26661618	both CNC-derived mesenchymal cells and epithelial cells in zebrafish (27908786). Oral, nasal, tongue, facial epithelium, tongue, tooth bud at E14.5 in mice (20549736)
67	*Wnt5a*	14	wingless-type MMTV integration site family, member 5A	Global-ENU-induced-point mutation. Homozygous	25807483 (global KO project. no information is available in MS, only in MGI)	CNC-derived mesenchymal cells (10021340)
68	*Zic3*	X	zinc finger protein of the cerebellum 3	Global-KO-KI-LacZ (*Zic3*$^{LacZ/LacZ}$ and *Zic3*$^{LacZ/Y}$), homozygous and *Sox2-Cre;Zic3*$^{F/Y}$, hemizygous	23184148	neural tube, migrating CNC cells, and CNC-derived mesenchymal cells (39424998)
**Spontaneous mice**						
1	*Apaf1*	10	apoptotic peptidase activating factor 1	Spontaneous-hypomorph (*Apaf1*$^{fog/fog}$). Homozygous	23892366; 9216040	Expressed in the brain at E9.5 (9753321)
2	*Br*	NA	NA	Radiation-induced. Homozygous	7833333; 8763472; 16533318	
3	*Lgl*	NA	NA	Transgenic-deletion. Homozygous	3406741; 2313245	
4	*Pgap2* (aka *Clpex*)	7	post-GPI attachment to proteins 2	Global ENU-induced-hypomorph. Homozygous	21515572; 31232685	Expressed in the mesenchyme (31232685)
5	*Ph*	NA	NA	Spontaneous and radiation-induced. Homozygous	Rasberry and Cattanach, 1994 Mouse Genome, 92(3):504-505; 10036976	
6	*Rgsc566*	NA	NA	Global-ENU-induced-KO. Heterozygous	No MS and image is available. Open database from RIKEN.	
7	*Rpl38*	11	ribosomal protein L38	Spontaneous (Ts/+ and Tss/+). Heterozygous	21529712; 10889952	CNC-derived mesenchymal cells (21529712)
8	*Tet1*	10	tet methylcytosine dioxygenase 1	Spontaneous KO. Homozygous	22246904	CNC-derived mesenchymal cells in zebrafish (38427557)
9	*Unicorn*	NA	NA	Global-ENU-induced. Homozygous. Missense mutations in *Raldh2* and *Leo1*	25807483 (global KO project. no information is available in MS, only in MGI); 31940751	
**Compound mutant mice**						first gene<br>second gene
1	*Aldh1a2* & *Aldh1a3*	9<br>7	aldehyde dehydrogenase family 1, subfamily A2<br>aldehyde dehydrogenase family 1, subfamily A3	*Aldh1a2*$^{-/-}$;*Aldh1a3*$^{-/-}$	17184764	RA signaling activities are found in mesenchymal- or mesodermal cells at the frontonasal process at E9.5. junction of the LNP and MNP and maxillary processes, eye, forebrain at E11.5 (26278034; 39870438; 9106168)
2	*Alx1* (aka *Cart1*) & *Alx4*	7	aristaless-like homeobox 1	*Alx1*$^{-/-}$;*Alx4*$^{-/-}$, *Alx1*$^{+/-}$;*Alx4*$^{-/-}$, and *Alx1*$^{-/-}$;*Alx4*$^{+/-}$	9847249	strong expression at the LNP and MNP and weak expression at the distal region

						of the maxillary process at E10.5 (35127681)
		10	aristaless-like homeobox 4			CNC-derived mesenchymal cells at E11.5 (15198690)
3	*Alx3* & *Alx4*	3	aristaless-like homeobox 3	*Alx3*$^{-/-}$;*Alx4*$^{+/-}$, *Alx3*$^{+/-}$;*Alx4*$^{-/-}$, and	11641221	CNC-derived mesenchymal cells in the frontonasal process at E9.5 (20534379)
		2	aristaless-like homeobox 4	*Al3*$^{-/-}$;*Alx4*$^{-/-}$		
4	*Axin1* & *Ctnnb1*	10	ALX homeobox 1	*Axin1*$^{\Delta C6/\Delta C6}$;*Ctnnb1*$^{+/-}$	19204372	ubiquitous
		9	catenin (cadherin associated protein), beta 1			
5	*Bbs7* & *Ift88*	3	Bardet-Biedl syndrome 7 (human)	*Bbs7*$^{-/-}$;*Ift88*$^{-/-}$	22228099	ubiquitous
		14	intraflagellar transport 88			
6	*Disp1* & *Shh*	1	dispatched RND transporter family member 1	*Shh-Cre*$^{/+}$; *Disp1*$^{\Delta 2/\Delta}$*2C*, *Shh-Cre*$^{/+}$; *Disp1*$^{C829F/\Delta 2C}$, *Dips1* $^{\Delta 2/\Delta 2}$;*Shh*$^{+/-}$, and *Dips1*$^{C829F/\Delta 2}$;*Shh*$^{+/-}$	15576405	unknown
		5	sonic hedgehog			
7	*Dlx5/6*	6	distal-less homeobox 5	Global KO. Homozygous	12193642; 12000792	Expressed in the ectomesenchyme of the first branchial arch of mandible and the ectoderm of frontonasal prominence at E9.0. The ectomesenchyme of the first branchial arch of the mandible and the ectoderm of inner nasal pit at E10.5 (10433912)
		6	distal-less homeobox 6			
8	*Gli2* & *Gli3*	1	GLI-Kruppel family member GLI2	*Wnt1-Cre*;*Gli2*$^{F/F}$;*Gli3*$^{F/F}$	27802276	strong expression in CNC-derived mesenchymal cells and weak expression in the epithelium. (22178118)
		13	GLI-Kruppel family member GLI3			strong expression in CNC-derived mesenchymal cells and weak expression in the epithelium at E11.5 and strong expression in the epithelium and weak expression in the mesenchyme at E14.5 (22178118)
9	*Gli2* & *Kif3a*	1	GLI-Kruppel family member GLI2	*Wnt1-Cre*;*Kif3a*$^{F/F}$;*dNGli2* (constitutive active GLI2A)	27802276	
		11	kinesin family member 3A			
10	*Insig1* & *Insig2*	5	insulin induced gene 1	*Insig1*$^{-/-}$;*Insig2*$^{-/-}$	16955138	ubiquitous
		1	insulin induced gene 2			
11	*Ndst1* & *Ndst3*	18	N-deacetylase/N-sulfotransferase (heparan glucosaminyl) 1	*Ndst1*$^{-/-}$;*Ndst3*$^{-/-}$	18385133 (no image is available, only text)	Expressed in the forebrain and the frontonasal process at E11.5 (16020517)
		3	N-deacetylase/N-sulfotransferase			Expressed in trigeminal neural crest cells and a part

			(heparan glucosaminyl) 3			of the brain at E10.5-E12.5. (18385133)
12	*Nuak1* & *Nuak2*	10	NUAK family, SNF1-like kinase, 1	*Nuak1*$^{-/-}$;*Nuak2*$^{-/-}$	22689267	Expressed in the ectoderm of the first branchial arch of the maxilla at E9.5. (22689267; 16715502)
		1	NUAK family, SNF1-like kinase, 2			Expressed in the ectoderm of the forebrain and frontonasal processes at E9.5. (22689267; 16715502)
13	*Pax3* & *Pax7*	1	paired box 3	*Pax3*$^{GFP/GFP}$;*Pax7*$^{LacZ/LacZ}$	25800090	
		4	paired box 7			CNC-derived cells in the frontonasal prominence and craniofacial muscle precursors at HH16-24 in chick (26042028). CNC-derived cells in the frontonasal prominence and the first branchial arches at E9.5 in Pax7-Cre;R26R lacZ tracing embryos (22848431)
14	*Pdgfra* & *Plekha1*	5	platelet derived growth factor receptor, alpha polypeptide	*Pdgfra*$^{+/-}$;*Plekha1*$^{-/-}$	17143286	
		7	pleckstrin homology domain containing, family A (phosphoinositide binding specific) member 1			unknown
15	*Pdgfra* & *Pdgfrb*	5	platelet derived growth factor receptor, alpha polypeptide	*Wnt1-Cre;Pdgfra*$^{F/F}$;*Pdgfrb*$^{F/F}$	17499702 (no image is available, only described in text)	
		18	platelet derived growth factor receptor, beta polypeptide	*Wnt1-Cre;Pdgfra*$^{PI3k/PI3k}$;*Pdgfrb*$^{F/F}$	27856617	CNC-derived mesenchymal cells (27856617)
16	*Rara* & *Rarg*	11	retinoic acid receptor, alpha	*Rara*$^{-/-}$;*Rarg*$^{-/-}$ and *Rara1*$^{-/-}$;*Rara2*$^{-/-}$;*Rarg*$^{-/-}$	7607067; 7626396	
		15	retinoic acid receptor, gamma			CNC-derived mesenchymal cells at the frontonasal prominence and the branchial arch at E9.5 and CNC cell derivatives at E12.5 (19471585)
17	*Tfap2a* & *Tfap2b*	13	transcription factor AP-2, alpha	*Wnt1-Cre;Tfap2a*$^{F/LacZ}$;*Tfap2b*$^{F/LacZ}$	29229773	Expressed in the frontonasal prominence and the first and second branchial arches at E9.0 (29229773)
		1	transcription factor AP-2 beta			
18	*Zeb1* & *Zeb2*	18	zinc finger E-box binding homeobox 1	*Zeb1*$^{LacZ/LacZ}$;*Zeb2*$^{+/-}$	16598713	CNC-derived mesenchymal cells at the frontonasal prominence and the first branchial arch at E9.5. expressed only in the neural tube at E11.5. (9389660)
		2	zinc finger E-box binding homeobox 1			Expressed in migrating CNC cells at E8.5. not expressed at E9.5 in CNC-derived mesenchymal cells. Re-expressed in CNC-derived

						mesenchymal cells after E11.5. (17478475)
**Compound mutant mice with vitamin A deficient diet**						
1	*Bco1* & *Rbp4*	8	beta-carotene oxygenase 1	*Bco1*$^{-/-}$;*Rbp4*$^{-/-}$	29892071	unknown
		19	retinol binding protein 4, plasma			not expressed
2	*Bco2* & *Rbp4*	9	beta-carotene oxygenase 2	*Bco2*$^{-/-}$;*Rbp4*$^{-/-}$	29892071	unknown
		19	retinol binding protein 4, plasma			

CNC, cranial neural crest; CL, cleft lip; CLP, cleft lip and palate; CP, cleft palate; CPO, cleft palate only; KO, knockout; MEE, medial edge epithelium; MS, manuscript.
